# Preliminary study on microR-148a and microR-10a in dermal papilla cells of Hu sheep

**DOI:** 10.1186/s12863-019-0770-8

**Published:** 2019-08-27

**Authors:** Xiaoyang Lv, Wen Gao, Chengyan Jin, Lihong Wang, Yue Wang, Weihao Chen, Shuangxia Zou, Sainan Huang, Zhifeng Li, Jinyu Wang, Wei Sun

**Affiliations:** 1 College of Animal Science and Technology, Yangzhou University, Yangzhou, 225009 China; 2 Joint international research laboratory of agriculture and agri - product safety of Ministry of Education of China, Yangzhou University, Yangzhou, 225009 China

**Keywords:** Hu sheep, miR-148a, miR-10a, *BMP7*, Dermal papilla cell

## Abstract

**Background:**

Hu sheep, a unique Chinese breed with high reproductive performance, are also well known for their rare white lambskin in China. The quality of lambskin is affected by hair follicles, and dermal papilla cells are an important component of hair follicles that plays a key role in hair follicle growth and development. This study helps elucidate the effect of miR-148a and miR-10a on hair follicle growth and development.

**Results:**

Based on the results of gene chip and high-throughput sequencing, bone morphogenetic protein 7 (*BMP7*) was used as a research object. Bioinformatics analysis and the dual-luciferase reporter system indicated that, along with Western blot and quantitative real-time polymerase chain reaction (qRT-PCR) that miR-148a and miR-10a target relationships with *BMP7*. *BMP7* was the target gene both for miR-148a and miR-10a by the dual-luciferase reporter system and Western blot. Hu sheep dermal papilla cells were successfully isolated and purified, and after transfecting miR-148a/miR-10a mimics and inhibitors into dermal papilla cells, a Cell Counting Kit-8 (CCK-8) was used to determine that miR-148a/miR-10a inhibited the proliferation of Hu sheep dermal papilla cells. In addition, after the overexpression of miR-148a, the expression levels of *Smad3* (*P* < 0.05)*, Smad6* (*P* < 0.05), *Smad4* (*P* < 0.01), and *Smad5* (*P* < 0.01) were significantly higher than those of the control groups. After the inhibition of miR-148a, the expression levels of *Smad3* (*P* < 0.05), *Smad4* (*P* < 0.05), and *TGF-β* (*P* < 0.01) were significantly lower than those of the control groups. After the overexpression of miR-10a, the expression levels of *Smad1* (*P* < 0.01)*, Smad2* (*P* < 0.05), *Smad4* (*P* < 0.01), *Smad5* (*P* < 0.01), and *TGF-β* (*P* < 0.05) were significantly lower than those of the control groups. After the inhibition of miR-10a, the expression levels of *Smad1* (*P* < 0.01) and *Smad2* (*P* < 0.05) were significantly lower than those of the control groups.

**Conclusions:**

These results revealed the target relationship between miR-148a, miR-10a and *BMP7*, and the effect of miR-148a and miR-10a on the proliferation of dermal papilla cells. They will provide the basis for a follow-up study on how miR-148a, and miR-10a mediate *BMP7* regulation of hair follicle growth and development.

## Background

The lambskin of Hu sheep is affected by various factors, and the type of pattern on the skin is one of the most important indicators of Hu sheep lambskin quality. A small wave pattern indicates higher quality than that of medium and large wave patterns [1, 2]. The density, fineness, and curvature of the wool are the key factors determining the quality of lambskin. These traits may be regulated by different candidate genes in the epidermis, such as *BMP7*, *β-catenin* and *Eda/Edar*, which affect the structural characteristics of the hair phenotype [3–5]. To explore the manifestation of hair phenotypes, it is necessary to study the growth and development of hair follicles, which are composed of various types of cells [6, 7]. The dermal papilla cells provide the necessary stimulation signals for the formation of hair follicles and the growth of hair shafts [8], which also control the size and shape of hair through regulating the number of hair cells [9]. The growth and development of hair follicles is dependent on dermal papilla cells to provide important nutrients and signal regulators [10, 11]. The dermal papilla cell not only regulates hair follicle growth and development, but also is considered as a versatile stem cell [12].

Mammalian hair follicles undergo a unique process of occurrence and differentiation in both embryonic and birth stages. Many signaling pathways are involved, but the signal cascade is not completely clear. Self-renewal and periodic development of hair follicles are regulated by synergistic or antagonistic effects of signaling molecules consisting of related factors and receptors in epidermal and mesenchymal cells [13]. This process also involves a number of signal transduction pathways that regulate the interaction of epidermal and mesenchymal components.

Currently, many of studies have shown that the transforming growth factor (TGF)-β/Smads, Wnt, Notch, and Shh signaling pathways are involved in the regulation of hair follicle growth and development [14–18], among which the TGF-β/Smads and Wnt signaling pathways have been confirmed to participate in the regulation mechanism of hair follicle differentiation and formation. Most members of the *BMP* family belonging to the *TGF-β* superfamily are the important signaling molecules involved in hair follicle development [19]. *TGF-β* plays an important role in regulating cell growth and differentiation. Furthermore, it is crucial to hair follicle development and formation. *Smads* is a unique signal factor in the TGF-β/Smads signaling pathway. When the corresponding receptor on the cell membrane surface binds to *TGF-β1*, *Smads* is responsible for the transmission of the TGF signal from the receptor into the nucleus [20]. Studies have shown that *Smads* exerts regulatory effects on hair follicle development, periodic growth and pigmentation [21, 22]. *BMP7*, one of the most biologically active proteins in the BMP family, is also an important signaling molecule involved in hair follicle development.

MicroRNAs (miRNAs) are a class of short (18–24 nt) single-stranded non-coding RNAs that can mediate gene silencing at the post-transcriptional level. As shown in recent studies, miRNAs are widely participate in the occurrence and periodic growth of hair follicles. The miRNAs in various types of hair follicle cells constitute an extremely complex network regulation system under the interaction of numerous regulatory factors and signaling pathways so that they can regulate hair follicle morphogenesis and periodic development [23, 24]. Ma et al. [25] screened 10 differentially expressed hair follicle-related miRNAs by transcriptome profiling, and the dual-luciferase reporter system verified that let-7a negatively regulates c-Myc and FGF5. Let-7a was involved in the regulation of hair follicle growth and development.

As illustrated from other study [26], miR-214 directly targets EZH2 to affect the expression of β-catenin and TCF-4 in the Wnt/β-catenin signaling pathway. MiR-214 can be considered as a candidate miRNA in the process of hair follicle development, and miR-399-5p [27], miR-195-5p [28] and miR-200 [29] are associated with hair follicle growth and development. Bai et al. [30] revealed that miR-148a-3p was significantly upregulated at the categen and telogen stages. MiR-148a-3p was enriched in many pathways and is involved in hair follicle growth and development. Yang et al. [31] found that miR-148b was highly expressed in the hair follicle growth phase and could promoted the proliferation of hair follicle cells. Moreover, miR-148b could also targeted NFAT5 and upregulated the expression of *β-catenin*, *cycD*, *c-jun* and *Wnt10b* to activate the Wnt/β-catenin signaling pathway. Although no direct studies have demonstrated that miR-148a is associated with the development of hair follicles, we hypothesized that because miR-148a is a member of the miR-148 family, it might also participate in the regulation of hair follicle growth and development in a manner similar to that of miR-148b. MiR-10a belongs to the miR-10/miR-100 family, and there have been many studies on the control that MiR-10a exerts during disease because of its location adjacent to the tumor-associated Hox gene family [32]. At present, it was found that miR-10a modulates the secretion of inflammatory cytokines and promotes the proliferation of promyelocytic leukemia cells [33, 34]. However, there are few reports on miR-10a and hair follicles. At present, miRNAs have been reported in the research on Hu sheep hair follicles, but they were only mentioned in the screening of differential miRNAs in hair follicles, indicating a lack of more in-depth research.

In this study, the effects of miR-148a and miR-10a on Hu sheep dermal papilla cell were explored, and the results will enrich the current research on hair follicle growth and development at the cellular and molecular levels. This research also provides a theoretical basis and new research ideas for improving the quality of Hu sheep lambskin.

## Results

### Isolation, culture and identification of Hu sheep dermal papilla cells

**Fig. 1:**
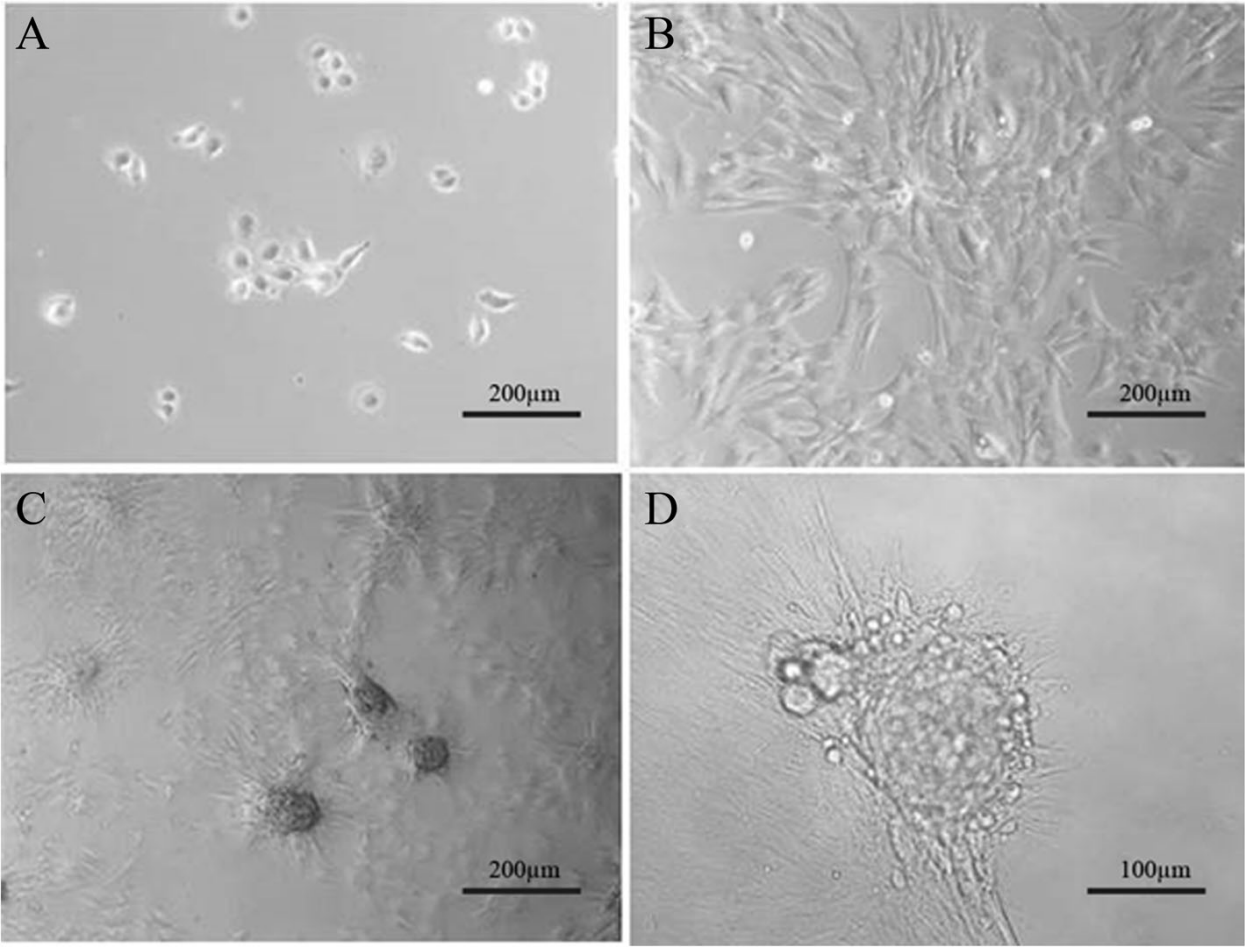
Sheep dermal papilla cells. **a**, **b**, and **c** show the cell morphology on the 2nd, 6th, and 12th days of cultivation (50×). **d** is the cell morphology on the 12th day of cultivation (100×)

The Hu sheep dermal papilla cells were separated by centrifugation combined with neutral protease and type IV collagenase digestion. They were observed to have a circular or elliptical shape under an inverted fluorescence microscope. On the day after cell isolation and culture, individual cells were observed under a digital microscope (Fig. 1a). Within one week, 85% of cells adhered to the Petri dish, and the cell proliferation rate was significantly accelerated. The cell body was large with a triangular or polygonal shape (Fig. 1b). The dermal papilla cells presented a radial growth trend 6 days later, and the cells gradually lost their original shape and formed a dense region (Fig. 1c). After 12 days, the cells gradually merged into cell clumps, and the cells around the clumps grew radially outward.

**Fig. 2:**
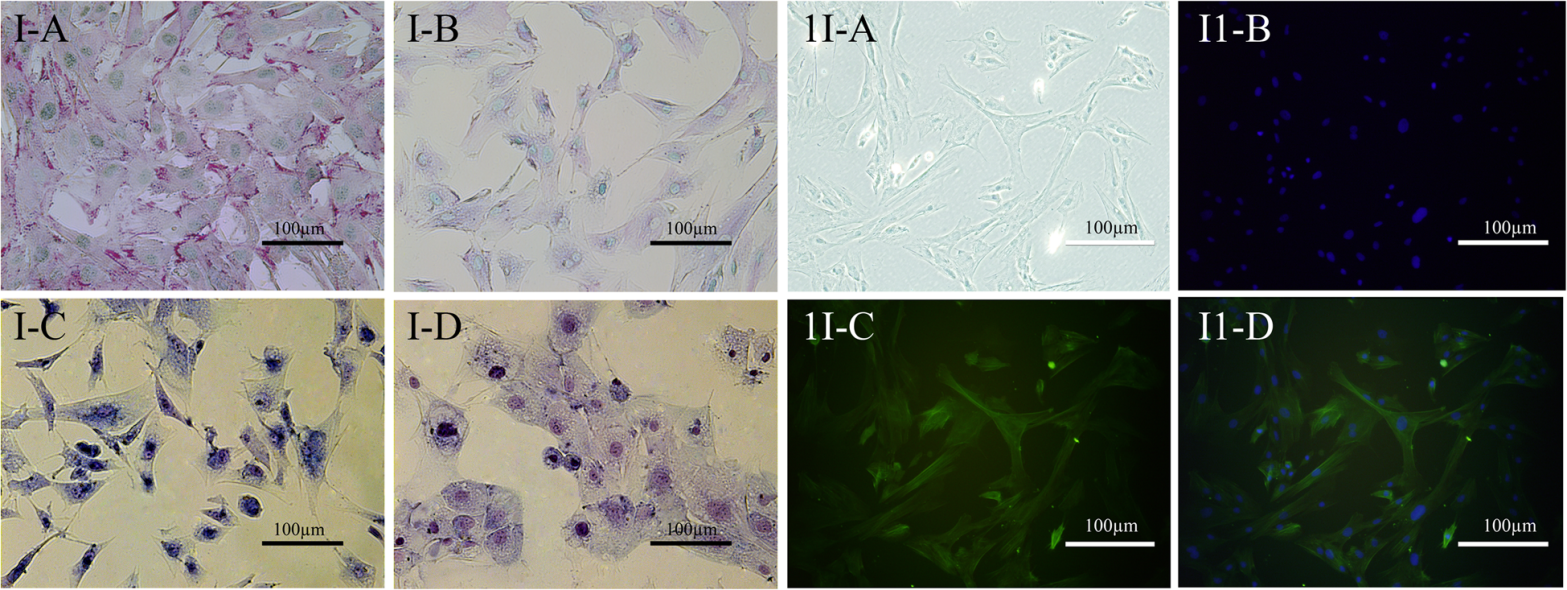
The identification of dermal papilla cells. I-A: PAS stain (100×), I-B AB-PAS stain (100×), I-C: toluidine blue stain (pH = 0.5, 100×), I-D: toluidine blue stain (pH = 6.0, 100×); II-A: light microscopy image of dermal papilla cells, II-B: nuclear staining with 4′,6-diamidino-2-phenylindole (DAPI), II-C: The immunofluorescence results for dermal papilla cells, II-D: merge image of II-B and II-C

The cells in the central region appeared to be multi-layered and they accumulated. They showed a tendency of growth with agglutination (Fig. 1d). This characteristic was still retained when the Hu sheep dermal papilla cells reseached the 20th generation. PAS staining revealed that the cytoplasm was different shades of purple, the nuclei were pale blue, and the edges of the dermal papilla cells were dark purple (Fig. 2 I-A). After staining with AB-PAS, the dermal papilla cell cytoplasm was fuchsia and the nuclei were cyan (Fig. 2 I-B). After toluidine blue staining, the dermal papilla cells showed metachromia, including dark blue (pH = 5) (Fig. 2 I-C) and fuchsia (pH = 6) (Fig. 2 I-D). Finally, immunofluorescence staining was used to detect the expression of α-smooth muscle actin (α-SMA), a specific marker of dermal papilla cells. Examination with a fluorescence inverted microscope revealed that the nuclei of dermal papilla cells were stained blue, and the cytoplasm was stained green (Fig. 2 II). It was confirmed that the cells in this study were Hu sheep dermal papilla cells.

### Dual-luciferase assay between miR-148a, miR-10a and *BMP7*

**Fig. 3:**
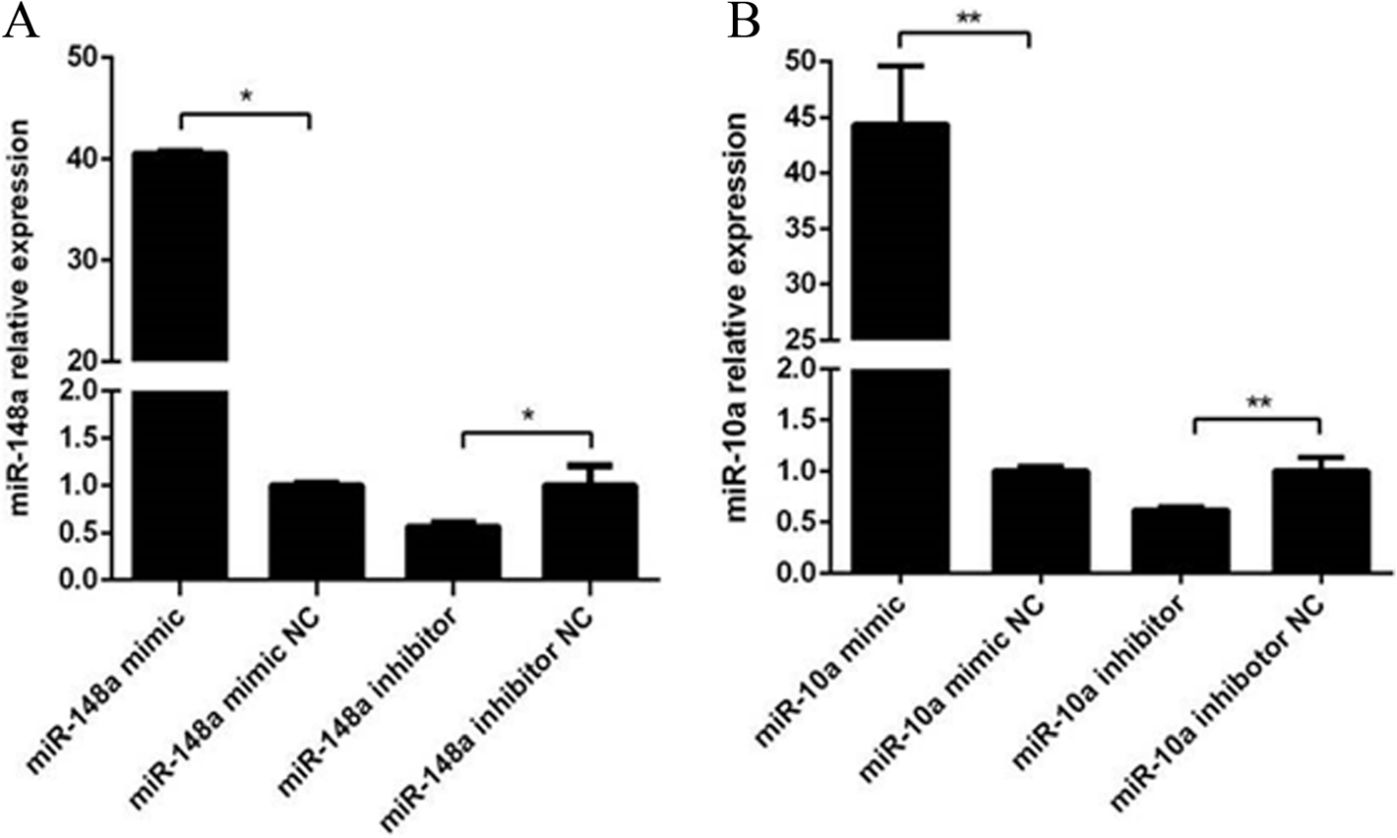
The relative expression of miR-148a and miR-10a (*n* = 3). **a** The relative expression of miR-148a in Hu sheep dermal papilla cells after overexpression or inhibition by miR-148a mimic or miR-148a inhibitor, **b** The relative expression of miR-10a after overexpression or inhibition by miR-10a mimic or miR-10a inhibitor. * represents a significant difference between the experimental group and the control group (*P* < 0.05). ** represents a great significant difference between the experimental group and the control group (*P* < 0.01)

First, we verified the transfection effects of miR-148a and miR-10a mimics and inhibitors. The mimic/mimic-NC and inhibitor/inhibitor-NC of miR-148a and miR-10a were transfected into Hu sheep dermal papilla cells to determine their effects in dermal papilla cells. The results showed that the expression levels of miR-148a and miR-10a increased in the cells after miR-148a mimic/mimic-NC, and miR-10a mimic/mimic-NC were transfected into dermal papilla cells, while the expression levels were inhibited after transfection of miR-148a inhibitor/inhibitor-NC and miR-10a inhibitor/inhibitor-NC. Therefore, miRNA mimics and inhibitors could be used in subsequent experiments (Fig. 3).

**Fig. 4:**
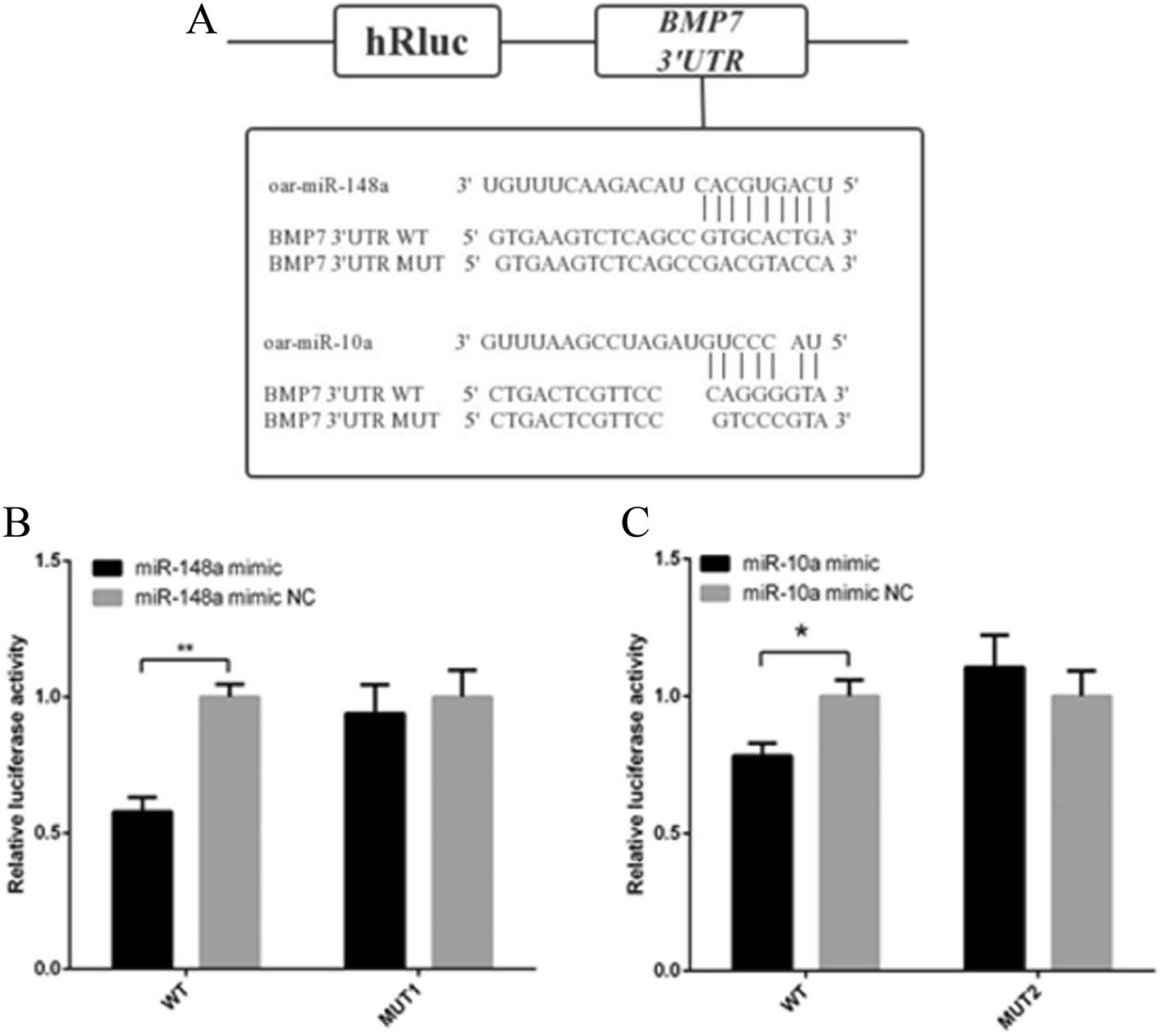
The result of the dual-luciferase reporter assay for target confirmation (n = 3). **a** Target site for miR-148a/miR-10a in the *BMP7* 3’UTR and the construction of the luciferase (Luc) expression vector fused with the *BMP7* 3’UTR. WT and MUT represent the Luc reporter vector with the WT, and the mutation at the miR-148a/miR-10a site in the *BMP7* 3’UTR, respectively. **b** The relative luciferase activity after co-transfecting miR-148a mimic/mimic-NC and the wild type (WT) vector or the mutant vector 1 in Hu sheep dermal papilla cells. **c** The relative luciferase activity after co-transfecting miR-10a mimic/mimic-NC and the WT type vector or the mutant vector 2 in Hu sheep dermal papilla cells. * represents a significant difference between the experimental group and the control group. ** represents a great significant difference between the experimental group and the control group

According to target gene prediction via Findtar3 (http://bio.sz.tsinghua.edu.cn/), RNA22 (http://cbcsrv.watson.ibm.com/rna22.htmL), miRBase (http://www.mirbase.org/) and miRanda (http://www.microrna.org/microrna/home.do) bioinformatics online software, we predicted the potential target miRNAs of *BMP7*. Through the intersection of the prediction and the miRNA sequencing results of the different patterns, two possible miRNAs (miR-148a, miR-10a) were selected as candidate miRNAs. The dual-luciferase reporter assay detected the fluorescence activity after transfection of miRNA mimics and inhibitors in Hu sheep dermal papilla cells for 36 h. The results showed that the relative luciferase activity in the WT group (miR-148a mimic + WT) was significantly lower than that in the control group (miR-148a mimic-NC + WT) (*P* < 0.01). The luciferase activity was not significantly different between the MUT1 group (miR-148a mimic+MUT1) and the control group (miR-148a mimic-NC + MUT1) (*P* > 0.05) (Fig. 4b). The luciferase activity in the WT group (miR-10a mimic + WT) was significantly lower than that in the control group (miR-10a mimic-NC + WT) (*P* < 0.05), while the luciferase activity in the MUT2 group (miR-10a mimic + MUT2) was not significantly different as compared to the control group (miR-10a mimic-NC + MUT2) (*P* > 0.05) (Fig. 4c). These results showed that there was binding between the seed regions of miR-148a and miR-10a and the 3’UTR region of *BMP7*.

### MiR-148a and miR-10a affected *BMP7* expression at the mRNA and *BMP7* protein levels

**Fig. 5:**
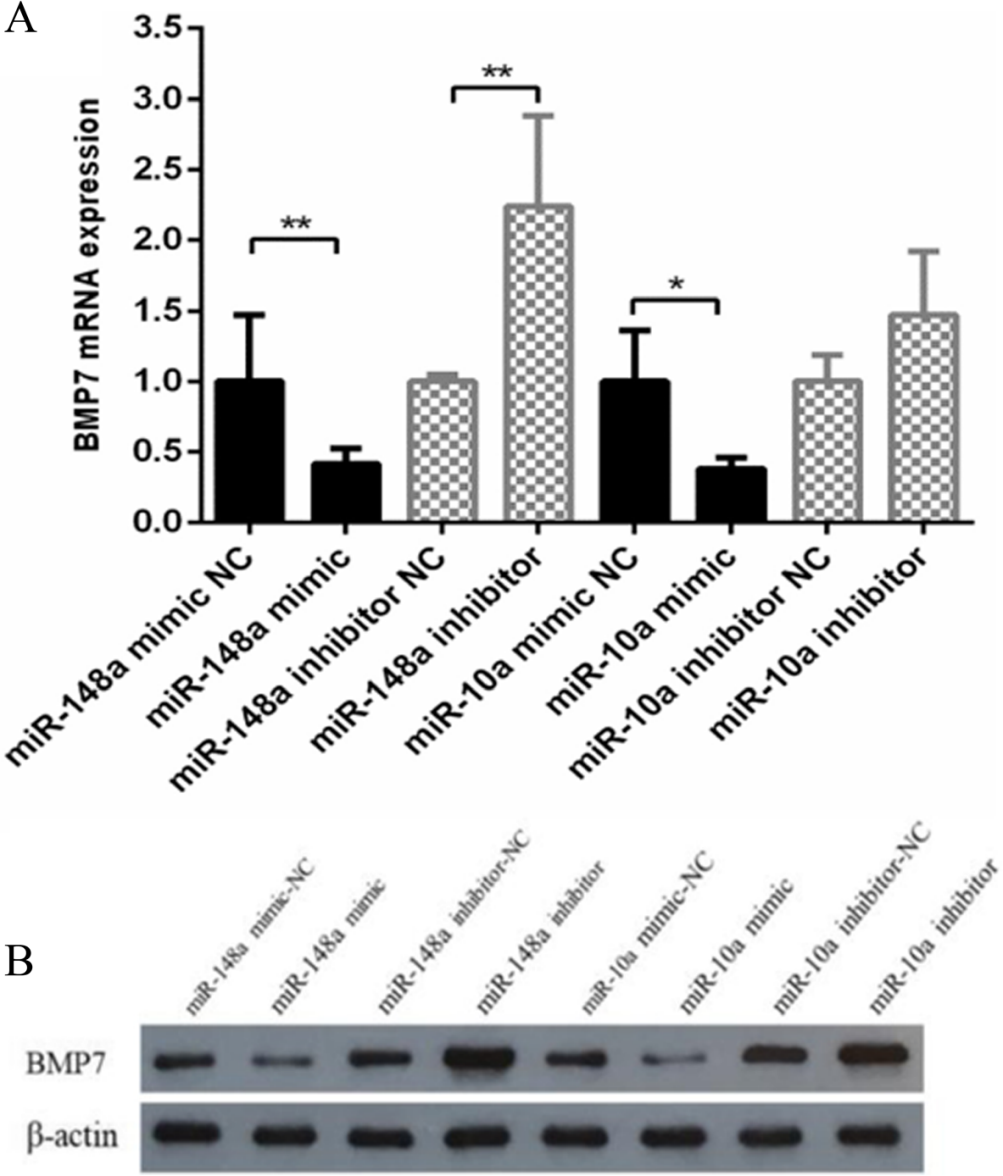
The expression levels of *BMP7* after overexpression and inhibition of miR-148a/miR-10a in dermal papilla cells (n = 3). **a** The mRNA expression of *BMP7*. **b** The protein expression of *BMP7*. * represents a significant difference between the experimental group and the control group. ** represents a great significant difference between the experimental group and the control group

To further validate the target relationship between *BMP7* and miR-148a/miR-10a, we examined the expression of *BMP7* mRNA and protein after overexpression and inhibition of miR-148a and miR-10a in dermal papilla cells. The results showed that the expression of *BMP7* was far lower than that of the control group after overexpression of miR-148a (*P* < 0.01), and it was far higher than that of the control group after inhibiting miR-148a (*P* < 0.01). The expression of *BMP7* was significantly lower than that of the control group after overexpression of miR-10a (*P* < 0.05), and it was higher than that of the control group after inhibiting miR-10a (*P* > 0.05). Although the difference was not significant, the expression of miR-10a in the inhibition group was still increased (Fig. 5a).

After overexpression of miR-148a and miR-10a in dermal papilla cells, the expression of BMP7 protein was lower than that of the negative control group. However, after inhibiting miR-148a and miR-10a, the expression of BMP7 protein was higher than that of the negative control group (Fig. 5b). The results showed that both miR-148a and miR-10a inhibited the expression of BMP7 protein, which further confirmed that *BMP7* is a target gene of miR-148a and miR-10a.

### Effects of miR-148a and miR-10a on proliferation of Hu sheep dermal papilla cells

**Fig. 6:**
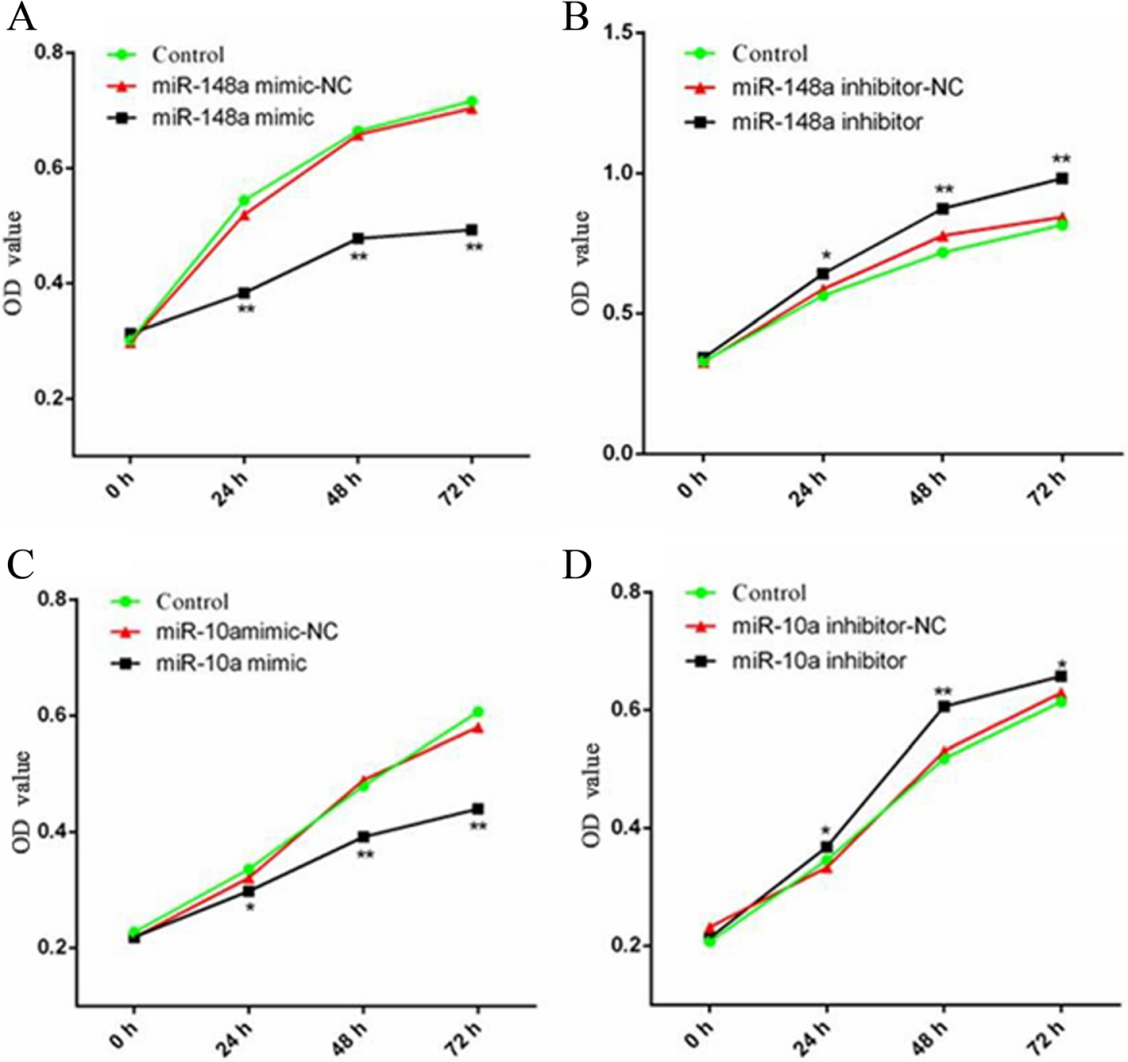
The results of Hu sheep dermal papilla cell proliferation with the CCK-8 detection assay (n = 3). **a** The proliferation of Hu sheep dermal papilla cells after the overexpression of miR-148a. **b** The proliferation of Hu sheep dermal papilla cells after the inhibition of miR-148a, **c** The proliferation of Hu sheep dermal papilla cells after the overexpression of miR-10a, **d** The proliferation of Hu sheep dermal papilla cells after the inhibition of miR-10a. * represents a significant difference between the experimental group and the control group (*P* < 0.05). ** represents a great significant difference between the experimental group and the control group (*P* < 0.01)

The growth rate of dermal papilla cells was detected using CCK-8 (No.CK04, Dojindo, Kumamoto, Japan) after transfection of miR-148a/miR-10a mimics and inhibitors. Compared with the negative control group, the proliferation rate of dermal papilla cells transfected with miR-148a mimic and miR-10a mimic for 24, 48, and 72 h was significantly decreased (*P* < 0.05), while the proliferation of dermal papilla cells transfected with miR-148a inhibitor and miR-10a inhibitor for 24, 48, and 72 h was significantly higher than that of the negative control group (*P* < 0.05) (Fig. 6). It was observed that both miR-148a and miR-10a inhibited the proliferation of Hu sheep dermal papilla cells.

### Effects of miR-148a and miR-10a on major genes in the TGF-β/Smads pathway

**Fig. 7:**
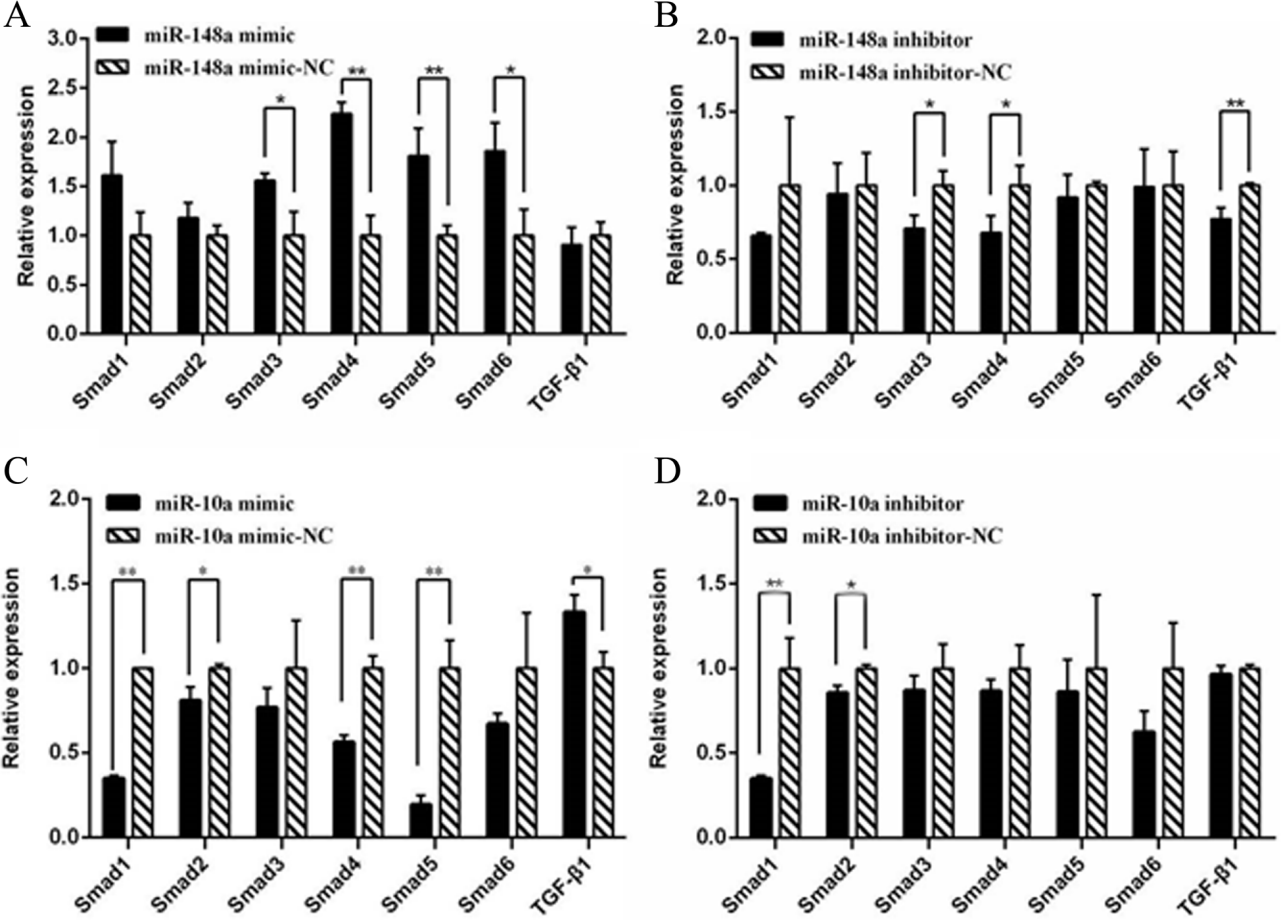
The results of gene expression in the TGF-β/Smads signaling pathway (n = 3). **a** The expression of different genes after miR-148a overexpression. **b** The expression of different genes after miR-148a inhibition. **c** The expression of different genes after miR-10a overexpression, **d** The expression of different genes after miR-148a inhibition. * represents a significant difference between the experimental group and the control group (*P* < 0.05). ** represents a great significant difference between the experimental group and the control group (*P* < 0.01)

The TGF-β/Smads pathway plays an important role in regulating the growth and development of hair follicles, and *BMP7* is involved in the TGFβ/Smads signaling pathway. To further explore the effects of miR-148a and miR-10a on the TGFβ/Smads signaling pathway, the key genes *Smad1*, *Smad2*, *Smad3*, *Smad4*, *Smad5*, *Smad6*, and *TGF-β1* in the TGFβ/Smads signaling pathway were detected by qRT-PCR after overexpression and inhibition of miR-148a and miR-10a. After overexpression of miR-148a, the expression of Smads genes was higher than that of the control group, among which, the *Smad3* and *Smad6* expression levels were significantly increased (*P* < 0.05), and those of *Smad4* and *Smad5* were significantly increased (*P* < 0.01). However, the expression level of *TGF-β1* was lower than that of the control group (Fig. 7a). After inhibiting miR-148a, the expression of Smads genes and *TGF-β1* was lower than that of the control group, and *Smad3* and *Smad4* expression levels were significantly decreased (*P* < 0.05), while there was an extremely significant difference between the expression of *TGF-β1* and the control group (*P* < 0.01) (Fig. 7b).

After overexpression of miR-10a, the expression of Smads genes in the experimental group was lower than that in the control group, and the expression of *Smad2* was significantly lower than that in the control group (*P* < 0.05). The expression of *Smad1*, *Smad4* and *Smad5* was far lower (*P* < 0.01), while the expression of *TGF-β1* was significantly higher than that of the control group (*P* < 0.05) (Fig. 7c). After inhibiting miR-10a, the expression of Smads and *TGF-β1* was lower than that in the control group *Smad1* was significantly lower (*P* < 0.05), and *Smad1* was even lower (*P* < 0.01) (Fig. 7d).

## Discussion

Currently, bioinformatics analysis is the most common method used to find target genes of miRNAs. We can calculate and filter the target gene or miRNAs targeting a specific gene by the interaction between the target gene and the miRNAs [35]. Common predictive software includes TargetScan, MiRBase, RNA22, PicTar, and Findtar3. Among them, RNA22 and Findtar3 are commonly used to predict miRNA and some specific species target points. In order to explore the *BMP7* target miRNAs of the sheep, RNA22 and Findtar3 were selected. Based on the results of high-throughput sequencing, miR-148a and miR-10a were selected for subsequent experimental studies.

Through complementation or incomplete complementation with the target mRNA, mature miRNAs direct the RNA-induced silencing complexes to degrade the target mRNA or inhibit translation of the target mRNA [36]. In order to further confirm that miR-148a/miR-10a targets *BMP7*, Western blot and qRT-PCR were used to detected *BMP7* mRNA and protein expression levels through overexpression and inhibition of miR-148a and miR-10a. It was found that both miR-148a and miR-10a directly bind to the targeted binding site in *BMP7* 3’UTR and promote the degradation of *BMP7* mRNA to reduce the BMP7 protein level. It has been confirmed that *BMP7* is a target gene of miR-148a and miR-10a, but the influence of miR-148a and miR-10a on Hu sheep dermal papilla cells requires further exploration.

The development of hair follicles is regulated by numerous signal factors. The interaction between different factors and different signal pathways constitutes an extremely complex regulatory network during the process of hair follicle growth. As an important regulator in the animal body, miRNA is also involved in the development and formation of hair follicles. There have been many reports on the function of miR-148a and miR-10a in cancer and oncogenesis, but few studies have been reported on hair follicles. Through overexpression and inhibition of miR-148a and miR-10a in dermal papilla cells, it was found that miR-148a and miR-10a inhibited the proliferation of dermal papilla cells. However, miR-148b can promote the proliferation of human dermal papilla cells and human hairy maternal cells. After analyzing the results of this study, it was determined that miR-148a and miR-148b were members of the same family, but they had different effects on the proliferation of dermal papilla cells. This might be caused by a variety of factors, such as structural differences between miR-148a and miR-148b or the different growth periods of the hair follicles during the detection. MiRNAs even have different effects on hair follicles in different species.

Previous studies have shown that some members of the *BMP* family play an important role in the regulation of hair follicle development, and the expression levels are different at different stages [37]. In this family, *BMP2* and *BMP4* are believed to have the effect of inhibiting the development of hair follicles [38–41]. Interestingly, by overexpressing and inhibiting miR-148a and miR-10a, we found that both miR-148a and miR-10a inhibited the proliferation of Hu sheep dermal papilla cells. Combining these data with the results of the dual-luciferase reporter assay, we speculated that *BMP7* may promote the proliferation of papilla cells, but the specific mechanism requires further study. In addition, there were reports that *BMP7* was highly expressed when the hair follicle was in the growth phase, especially when the hair follicle cells started to proliferate at the basement membrane. When the hair follicle entered the degenerative phase or the resting phase, *BMP7* was low or even not expressed [42]. This indicates that *BMP7* may promote the proliferation of hair follicle cells in the hair follicle growth phase. However, Wang [43] reported that *BMP7* has an inhibitory effect on the growth of wool length in fine wool sheep. This is not inconsistent with the speculation of the current study, because this study collected 2-day-old Hu lamb hair follicles, and Wang used follicles from adult Aohan fine wool sheep. One potential explanation is that different sheep breeds, different cycles, and other factors will have different effects on the results. Some studies have revealed that miR-148 and miR-10 inhibited the proliferation of different types of cells. For example, miR-148-3p inhibited the growth of glioblastoma by targeting DNMT1 and PCa cell growth in vivo [44, 45]. It was also found that miR-148a could suppress human renal cell carcinoma malignancy by targeting AKT2 [46]. Additionally, miR-10a was found to inhibit cell proliferation by targeting BCL6 in diffuse large B-cell lymphoma [47]. MiR-10a-5p was proved to have the capacity to inhibit keratinocyte proliferation [48]. According to these reports, miR-148a and miR-10a can inhibit cell proliferation which is consistent with our results.

Studies have shown that *BMP7* and *TGF-β1* are mutually antagonistic. When miR-148a is overexpressed in Hu sheep dermal papilla cells, the expression level of *BMP7* will decrease relative to miR-148a targeting to *BMP7*. If there is no other condition, the expression of *TGF-β1* will be theoretically upregulated. However, *TGF-β1* expression was downregulated after overexpression of miR-148a. In the TGF-β/Smads pathway, *Smad6* and *Smad7* are the major negative regulators of the TGF-β/Smads signal transduction pathway. *Smad6* inhibits the phosphorylation of *Smad2* and *Smad3* by binding to type I receptors activated by *TGF-β1*, thereby negatively regulating the signal transduction of *TGF-β1* [49]. We hypothesized that *Smad6* exerts a major influence in the overexpression and inhibition of miR-148a in Hu sheep dermal papilla cells, which involves inhibiting the phosphorylation of *Smad2* and *Smad3*, thereby impeding the signal transduction of *TGF-β1*. When overexpression or inhibition of miR-10a occurs, the expression of *Smad1* to *Smad6* is downregulated, although the opposite trend occurs for *TGF-β1* expression. We speculate that miR-10a could be associated with the TGF-β/Smads pathway, but it might also be related to other pathways.

## Conclusions

In summary, the results of this study to some extent reveal that miR-148a and miR-10a can inhibit the proliferation of Hu sheep dermal papilla cells, and they are associated with hair follicle growth and development. However, the growth and development of hair follicles is a delicate and extremely complex process. Therefore, follow-up studies are required to further reveal the mechanisms of hair follicle growth and development.

## Methods

### Isolation, culture and identification of Hu sheep dermal papilla cells

A lively and healthy 2-day-old Hu lamb was selected from a Suzhou stud farm in China, approximately 1 cm^2^ of the dorsal skin tissue was surgically removed, and the wound was then swabbed with iodine and bandaged with medical gauze. After disinfection for 15 s with 75% alcohol, the skin was washed with phosphate-buffered saline (PBS) containing a double antibody, and finally placed in Dulbecco’s modified Eagle’s medium (DMEM) containing penicillin and streptomycin to separate and culture the dermal papilla cells. Using a sterile surgical blade to remove some obvious fat and connective tissue on the surface, the tissue was cut into a 1-mm^2^ block, and the dermal papilla cells were cultured by neutral protease and collagenase digestion. According to the characteristics that different cells have different sensitivities to the enzymes, the dermal papilla cells were subcultured and purified by differential centrifugation. Finally, the dermal papilla cells were identified by periodic acid Schiff (PAS), Alcian blue-periodic acid Schiff (AB-PAS), toluidine blue staining and immunofluorescence staining and nuclear staining with 4′,6-diamidino-2-phenylindole (DAPI).

### Dual-luciferase assay between miR-148a, miR-10a and *BMP7*

The 3′ untranslating region (3’UTR) sequence of the *BMP7* was amplified, and cloned into the pmiR-RB-REPORTTM dual-luciferase reporter vector using the Trelief SoSoo Cloning Kit (No. TSV-S1; TsingKe, Beijing, China) (Table 1). To construct two mutant vectors, oar-*BMP7*-MUT1 (TGCACTG was mutated to ACGTACC at 318–324 bp) and oar-*BMP7*-MUT2 (CAGGG was mutated to GTCCC at 334–338 bp) (Table 2), a Fast Site-Directed Mutagenesis Kit (No. KM101; Tiangen, Beijing, China) was used. Human embryonic kidney HEK293T cells were cultured in DMEM/Ham’s F12 nutrient mixture (F12) (Gibco, NY, US) supplemented with 10% fetal bovine serum (Sigma, Santa Clara, CA, US) with 5% CO_2_ in air at 37 °C. The HEK293T cells were cultured in 24-well plates to 80% confluence and transfected using FuGENE® HD transfection reagent (Promega, Madison, WI, US), according to the manufacturer’s instructions. MiRNA mimic/inhibitor was purchased from Guangzhou RiboBio Co.,Ltd. The experimental groupings were: miR-148a mimic + WT, miR-148a mimic-NC + WT, miR-148a mimic + MUT1, miR-148a mimic-NC + MUT1 and miR-10a mimic + WT, miR-10a mimic-NC + WT, miR-10a mimic + MUT2, miR-10a mimic-NC + MUT2. Three parallel experimental groups were set up for each sample. Dual-luciferase reporter assays were performed according to the Promega Dual-Glo Luciferase-Assay System reagent instructions.

**Table 1:** Amplification primers information of *BMP7* 3’UTR sequence

Name	Primer	Length/bp
*BMP7*-F(XhoI)	GCGGCTCGAGCTCTTCCTGGAAGTCACACGCTT	480
*BMP7*-R(NotI)	AATGCGGCCGCTTTTTTTTTTTTCATCATCGTTTTA	

Note: The underline is the enzyme cleavage site, and the sequence before underline is the protection base

**Table 2:** Mutation vector construction of *BMP7* 3’UTR

Name	Primer sequence
Oar-BMP7-MUT1-F	TCAGCCGACGTACCACTCGTTCCCAGGGTAA
Oar-BMP7-MUT1-R	GAACGAGTGGTACGTCGGCTGAGACTTCACAGC
Oar-BMP7-MUT2-F	CTCGTTCCGTCCCGTAATACGACACTCTTCA
Oar-BMP7-MUT2-R	GTAATTACGGGACGGAACGAGTCAGTGCACG

Note: The underline means mutation sequence

### qRT-PCR and Western blot

In order to further explore the influence of mir-148a and mir-10a on related genes in the TGF-beta /Smads signaling pathway, *Smad1*, *Smad2*, *Smad3*, *Smad4*, *Smad5*, *Smad6*, and *TGF-β1* were selected for qRT-PCR and western blot. According to the sheep gene sequence published in GenBank, Primer3web (http://primer3.ut.ee/) was used to design primers for *Smad1*, *Smad2*, *Smad3*, *Smad4*, *Smad5*, *Smad6*, and *TGF-β1* (Table 3). After the cells were transfected for 36 h, the total RNA was extracted using RNAsimple Total RNA Kit (Tiangen) and reverse-transcribed into cDNA using a FastQuant RT Kit (with gDNase) (Tiangen). The expression of *BMP7* mRNA in the different treatment groups was detected by qRT-PCR using TB Green Premix Ex Taq II (Tli RNaseH Plus) (TaKaRa, Dalian, China), according to the manufacturer’s instructions.

**Table 3:** Primers information

Genes	References sequences	Sequences (5′ → 3′)	Product length (bp)
*Smad1*	AY035385	F: GAAAGCCCCGTTCTTCCTCC R: GTTGGGCTGCTGGAAAGAAT	150
*Smad2*	AY185300.1	F: GCAGGAATTGAGCCACAGAG R: GCTGCAAATCCAAGCTCTGA	172
*Smad3*	AF508024.1	F: GGACGACTACAGCCATTCCA R: ATTCGGGGAGAGGTTTGGAG	172
*Smad4*	NM_001267886	F:CTTCAGGTGGCTGGTCGG R:TCCAGGTGATACAACTCGTTCA	177
*Smad5*	AF508027	F:CCAGTATATCCAGCAGAGATGTT R:AAGCTTCCCCAACACGATTG	102
*Smad6*	XM_004010255.3	F: CTGCTCGGACGCCTCTTC R: GGGTGGCGGTGATTCTGG	105
*TGF-β1*	NM_001009400	F:GGTGGAATACGGCAACAAAATC R:TGCTGCTCCACTTTTAACTTGA	162
*GAPDH*	NM_001190390.1	F: GTCGGAGTGAACGGATTTGG R: CATTGATGACGAGCTTCCCG	196

For the Western blot analysis, dermal papilla cells were lysed using radioimmunoprecipitation assay lysis buffer (Storng) (CWBIO, Beijing, China). The protein concentrations of different groups were measured with a bicinchoninic acid Protein Assay Kit (Beyotime, Jishou, China). The proteins were separated by SDS-PAGE, transferred to polyvinylidene difluoride membranes, and probed with 1:500 rabbit anti-BMP7 (Sigma, sab2107883, USA) and 1:1000 rabbit anti-β-actin (CWBIO) antibodies, and then with 1: 1000 polyclonal goat anti-rabbit IgG HRG conjugated antibodies (CWBIO). All steps were carried out in accordance with the instructions. Signals were detected with the enhanced chemiluminescence eECL Western Blot kit (CWBIO). Protein was detected and analyzed by the Wes automated Western Blot Analysis System (Protein Simple, CA, US).

### Overexpression and inhibition of miR-148a, miR-10a

The culture and transfection methods for Hu sheep dermal papilla cells were the same as those for HEK293T cells. The dermal papilla cells were transferred to 96 -well plates with 0.25% trypsin 36 h after transfection with miR-148a/miR-10a mimics and inhibitors. The medium was discarded after 0, 24,48 and 72 h, 10 μL CCK-8 solution was added to each well. The OD value was measured at 450 nm with a Tecan Infinite F200/M200 microplate reader (Tecan, Shanghai, China) after 2 h. At the same time, the expression of *Smad1*, *Smad2*, *Smad3*, *Smad4*, *Smad5*, *Smad6*, and *TGF-β1* was detected using qRT-PCR 24 h hours after transfection.

### Data analysis

The relative gene expression levels were analyzed by the 2^-ΔΔCT^ method. Independent sample *t* tests were performed using SPSS 16.0 software (* denotes *p* < 0.05, significant difference; ** denotes *p* < 0.01, extremely significant difference) [50], and drawing was performed using GraphPad Prism 6 software. Three biological replicates were used for each analysis, and all error bars in the results represent the mean ± SD.

## Data Availability

The data sets supporting the results of this article are included within the article.

## Abbreviations

AB-PAS: Alcian Blue-Periodic Acid Schiff
BMP7: Bone morphogenetic protein 7
CCK-8: Cell Counting Kit 8
DAPI: 4′, 6-Diamidino-2-Phenylindole
MiRNA: MicroRNA
PAS: Periodic Acid Schiff
qRT-PCR: Quantitative real-time polymerase chain reaction
TGF-β1: Transforming growth factor-β1
UTR: Untranslating Region
